# Genetic counseling certificate program: A program evaluation of undergraduate exposure to genetic counseling

**DOI:** 10.1002/jgc4.1564

**Published:** 2022-02-22

**Authors:** Erin McGraw, Jessica Rispoli, Michele B. Horner, Gary A. Heiman

**Affiliations:** ^1^ Department of Genetics and the Human Genetics Institute of New Jersey Rutgers, the State University of New Jersey Piscataway New Jersey USA; ^2^ Hackensack Meridian Jersey Shore University Medical Center Neptune New Jersey USA

**Keywords:** education, familiarity, genetic counseling, program evaluation, undergraduate students

## Abstract

Undergraduate genetic counseling exposure can generate interest in a growing field, help students prepare to apply to graduate‐level programs, and introduce underrepresented populations to the career. One form of exposure that currently exists is the Genetic Counseling Certificate Program (GCCP), which is offered to undergraduate students at Rutgers University. To determine the effectiveness, benefits, and limitations of the GCCP, a program evaluation was conducted. Former GCCP students were surveyed to assess how they perceived the program. Overall, most students thought the program successfully met its objectives and thought their participation in the GCCP was beneficial. Because it is viewed favorably by former students, implementing something similar to the GCCP may be an option for institutions looking to offer additional opportunities to their undergraduates. Not only could creating programs like the GCCP enhance undergraduates’ knowledge of the genetic counseling profession, but it could also contribute toward diversification of the field.

**What is known about this topic**

Exposing undergraduates to the genetic counseling career can generate more interest in an expanding profession and introduce a diverse set of individuals to the field. Types of undergraduate exposure can range from introductory genetic counseling lectures in science courses, to genetic counseling clubs, to more formalized offerings, like a genetic counseling certificate program or a minor.

**What this paper adds to the topic**

This paper describes the results of a program evaluation of the undergraduate Genetic Counseling Certificate Program (GCCP) at Rutgers University. The GCCP is a unique form of undergraduate genetic counseling exposure that is advantageous for several reasons, ranging from its ability to familiarize students with the profession to its effectiveness at introducing students from underrepresented populations to the field.

Both advancements in genetics and the continued integration of genomics into medicine have resulted in an increased need for genetics professionals, including genetic counselors (Gerard et al., 2018; Pan et al., 2016). Between 2019 and 2029, the genetic counseling profession is projected to grow by 21% (Bureau of Labor Statistics, 2020). Recently, in an effort to accommodate the need for more genetic counselors, genetic counseling graduate programs have started to accept more applicants, and several new graduate programs have been opened (Gerard et al., 2018; Wiesman et al., 2016).

To be competitive applicants, students who apply to genetic counseling graduate programs must meet a variety of requirements. Programs typically expect their applicants to have the following: undergraduate coursework in the sciences, acceptable GRE scores, letters of recommendation, advocacy experience working with individuals who are either in a crisis or have a genetic condition, and experience shadowing or interviewing a genetic counselor (Association of Genetic Counseling Program Directors, 2021). Before students consider applying to graduate programs, however, it can be helpful for them to have a good understanding of the genetic counseling profession. Unfortunately, many undergraduate students are unfamiliar with the career. Despite the fact over 75% of undergraduate students heard of genetic counseling, less than 10% said they were very familiar with it (Gerard et al., 2018). Since genetic counseling is not well understood by most undergraduates, offering exposure to the career in the undergraduate setting can familiarize students with the profession and generate interest in a growing field. Additionally, it can provide knowledge of application prerequisites, allowing students to pursue needed courses and experiences to become a competitive applicant.

Beyond introducing students to the field and preparing them to apply to graduate‐level programs, undergraduate exposure can increase interest from underrepresented populations. Currently, 95% of genetic counselors identify as female and 90% identified as white (National Society of Genetic Counselors, 2020). Despite this lack of diversity, students from underrepresented populations are just as likely to consider a career in genetic counseling, if given exposure to the career (Oh & Lewis, 2005). Unfortunately, when compared to their counterparts, these students have less exposure to the field. Data have suggested that exposing undergraduates to genetic counseling may improve minority students’ awareness of the career (Price et al., 2020).

One unique form of exposure being offered to undergraduate students is the Genetic Counseling Certificate Program (GCCP) at Rutgers University. The GCCP is intended for declared genetics majors who express an interest in becoming a genetic counselor. After taking two genetics core sequence courses, students can apply to the GCCP. Typically, students submit applications during their junior year. In addition to majoring in genetics, applicants must have a minimum 3.2 cumulative grade point average (>3.4 preferred). They also need to submit a 1–2 page essay describing their interest in the genetic counseling field. Upon application submission, each applicant is interviewed by the GCCP director. Students accepted into the GCCP must complete a series of didactic courses worth 15 credits (e.g., Genetic Counseling Rotation Course, abnormal psychology, statistics, and ethics) and a year‐long crisis volunteer experience. In the semester‐long Rotation Course, students are placed at a local genetic counseling clinic where they are given an opportunity to shadow practicing genetic counselors. Every week, students meet with the GCCP director to discuss the cases they have observed as well as the genetic counseling career as a whole. The GCCP director also meets with students to provide guidance with the graduate school application process.

To promote awareness of the GCCP and, subsequently, the genetic counseling career, it is advertised at various university events. These events include specific university lectures that are open to all students, various courses for both genetics majors and non‐majors, Rutgers Day, and the Rutgers University Genetic Counseling Master's Program's open house.

The GCCP has been offered to undergraduate students at Rutgers for ten years. Despite the fact that it has existed for a decade, a formal evaluation to determine effectiveness and overall satisfaction has not been performed. Therefore, the purpose of this study was to conduct an educational program evaluation of the GCCP by surveying former GCCP students. A second purpose was to evaluate how such an undergraduate program could increase diversity of those applying to and accepted into the graduate programs. By diversity, we refer to the expanded concept described by the National Society of Genetic Counselor's (NSGC) Diversity Special Interest Group that includes, among others, places of origin, language spoken, sexual orientation, disability status, and gender (Mittman & Downs, 2008).

Thirty‐four former GCCP students were eligible to participate in this program evaluation. Of the 34 former GCCP students, 27 (79.4%) applied to genetic counseling graduate programs, and, of these 27, 26 (96.3%) were accepted. Of the remaining seven students, six decided to pursue a different career. The final student was lost to follow‐up, and it was unknown if they applied to genetic counseling graduate programs.

Of the 34 eligible participants, 17 (50.0%) were from an underrepresented population in the genetic counseling field. Thirteen (76.5%) of these students chose to apply to genetic counseling graduate programs, and all thirteen (100%) were accepted. The remaining four students chose to pursue a different career. The above data were obtained from the GCCP director, who keeps track of prior GCCP students’ demographic information and career paths.

A survey was developed for this program evaluation (see supplementary Material B). It contained closed‐ended, open‐ended, check‐all‐that‐apply, and Likert scale questions. The survey assessed former GCCP students’ thoughts on the certificate program's objectives, strengths, and weaknesses. Additionally, it assessed how participation in the program affected students’ future career decisions.

The survey was created in Qualtrics. Individuals were contacted directly via email to ask for their participation in this program evaluation. All collected survey responses were anonymous. This study was approved by the Rutgers University Institutional Review Board.

Closed‐ended, check‐all‐that apply, and Likert scale questions were analyzed by calculating the percentage of participants who chose each response. Responses to open‐ended questions were reviewed by two evaluation team members (EM and GAH). Both individually reviewed responses for similar themes and grouped them accordingly. After independently grouping responses, the reviewers compared their results. Any discrepancies were discussed between them until a conclusion was reached. After grouping responses, the prevalence of each theme was calculated using percentages. Using these percentages, the two most common themes for each open‐ended question were determined.

Of the 34 GCCP students eligible to participate, valid emails were available for 33. Twenty‐three students completed the survey for a response rate of 69.7%. Of the 23 respondents, 47.8% (*n* = 11) reported that they were part of an underrepresented population (e.g., Hispanic, LGBTQ, and male). Ten respondents (43.5%) reported that they completed their undergraduate degree within the last five years.

There were three categories of former GCCP students: (a) applied and accepted into genetic counseling graduate programs (henceforth called ‘accepted students’), (b) applied and not accepted (henceforth called ‘not‐accepted students’), and (c) not applied (henceforth called ‘alternative students’). Of the 23 respondents, 18 (78.3%) were accepted students, one (4.3%) was a not‐accepted student, and four (17.4%) were alternative students.

Students were asked to indicate if the GCCP met its four objectives: (a) enhancing students’ understanding of the genetic counseling profession, (b) enhancing students’ understanding of the application requirements for graduate‐level programs, (c) fostering experience talking with people in a crisis, and (d) facilitating experience in clinical genetics settings. Overall, the majority (78.3%) of respondents indicated that all four objectives were successfully met.

Over 90% of respondents indicated they would recommend the GCCP to undergraduate students interested in genetic counseling, the amount of time they committed to the program was appropriate, and their participation in the GCCP was a positive experience. Slightly less than 70% indicated GCCP participation impacted their decision to apply to genetic counseling graduate programs. Four participants (17.4%), all of which were accepted students, indicated that GCCP participation did not help them decide whether or not they wanted to apply to genetic counseling graduate programs. Respondents indicated that the most beneficial aspect of the program was the clinical rotation course (see Supplementary Material A for student comments). The second most beneficial aspect was obtaining help with the application process by assuring the proper prerequisites had been completed and/or by prepping students for interviews.

Two themes emerged regarding feedback on how the GCCP could be improved. Respondents indicated that the GCCP should better educate its students about non‐traditional roles in genetic counseling. Additionally, rather than finding them on their own, participants suggested the GCCP could help its students find volunteer opportunities that allow them to work with individuals in a crisis.

Of the eighteen accepted students, 16 (88.9%) applied once prior to their acceptance, while the remaining two students (11.1%) applied twice before being accepted. Seventeen participants (94.4%) applied to graduate programs as a senior in college, and one (5.6%) applied after taking a gap year. When asked if participating in the GCCP impacted when they chose to apply, 11 (61.1%) students responded yes. Of these 11, seven (63.6%) indicated the GCCP prepared them to apply and enter graduate school, which is why they thought participating in the program impacted when they applied.

Seventeen accepted students (94.4%) thought participating in the program was advantageous to them during their time as a graduate student. Of these 17 students, over 70% said the program provided them with a good baseline understanding of the genetic counseling field, familiarized them with some of the basic skills genetic counselors regularly use (e.g., taking a family history), and/or exposed them to counseling techniques that could be applied to the genetic counseling profession. All 18 accepted students said that, as practicing genetic counselors, they would be willing to work with students participating in the GCCP or something similar.

The not‐accepted student applied to master's programs twice before deciding not to apply again. This student did not feel participating in the GCCP impacted when they chose to apply.

Half (2/4) of the alternative students said they would have applied to genetic counseling graduate programs had they not participated in the GCCP. Although they decided not to apply, all four respondents believed the certificate program influenced the career path they chose to pursue.

The GCCP at Rutgers University is a unique form of undergraduate genetic counseling exposure that familiarizes students with the profession and prepares them to apply to graduate‐level programs. One clear strength of the GCCP is the semester‐long clinical rotation course. This course provides students with a considerable amount of exposure to genetic counseling in a clinical setting and allows them to become more familiar with the various responsibilities of a genetic counselor. Considering many undergraduate students may face challenges securing shadowing opportunities on their own (limited counselor availability, few counselors in geographic area, etc.), the GCCP’s ability to secure a rotation for its students is invaluable.

In addition to the clinical rotation, another of the GCCP’s strengths lies in its ability to aid students with future career decisions. Most former GCCP students indicated that participating in the certificate program helped them decide if they wanted to apply to genetic counseling graduate programs. In fact, half of the alternative students said they would have applied had they not participated in the GCCP. Therefore, because the exposure the GCCP offers provides students with a better understanding of the profession, it can prevent some from pursuing a career that is not a good fit.

Of the 23 students who participated in this evaluation, only four (17.4%) indicated that GCCP participation did not help them decide whether or not they wanted to apply to genetic counseling graduate programs. Because all four of these respondents were accepted students, it is likely that, prior to participating in the certificate program, they were already certain they wanted to pursue a career in genetic counseling.

Another important aspect of the GCCP is that it has exposed students from a diverse background to the career. Half of the students (17/34) eligible to participate in this study were part of an underrepresented population in the genetic counseling field. This proportion differs from the demographics of practicing genetic counselors, where 90% of counselors identify as white, and 95% identify as female (National Society of Genetic Counselors, 2020). While only a small number of students have participated in the GCCP, the current assessment of its demographics suggests it has successfully introduced underrepresented populations to a field that lacks diversity. In addition to introducing underrepresented populations to the field, the GCCP has also contributed to their acceptance into genetic counseling graduate programs. Of the 17 GCCP students who were part of an underrepresented population, 13 (76.5%) chose to apply to graduate programs, and all 13 (100%) were accepted.

While the GCCP has many strengths, former students did suggest improvements, such as better educating students about non‐traditional roles available to genetic counselors. Non‐traditional roles are becoming more prevalent. According to NSGC’s PSS, 25% of counselors reported they had non‐direct patient care positions, while 23% reported having mixed direct and non‐direct patient care positions (National Society of Genetic Counselors, 2020). Because these non‐traditional roles are becoming more plentiful and popular, exposing students to them can assure they have a more comprehensive understanding of the genetic counseling profession.

Because it has reported value and contributes to the acceptance of underrepresented populations into graduate‐level programs, implementing something similar to the GCCP may be an important undertaking for other institutions. Not only would they be providing their undergraduates with exposure to genetic counseling, but they could also potentially contribute to the field's diversification. Each institution would be able to determine what requirements (science courses, major, GPA, etc.) make the most sense for their student body. It is important to acknowledge that institutions may face barriers if they choose to create GCCP‐like programs. While this survey did not assess potential barriers, one would anticipate time commitment, number of genetic counselors in the geographic location, little interaction with undergraduate students, ability to secure shadowing opportunities, etc. to play a role.

Our findings should be considered in the context of certain limitations. Because so few students completed the certificate program, only a small number of individuals were eligible to participate. The significance of the responses to these surveys must be taken into context with the small sample size. Additionally, because the sample size was small, participant anonymity had to be taken into consideration when the survey was created. Certain demographic information, such as participants’ specific ethnic background and gender, was not collected. Therefore, although it is known that 11 participants considered themselves part of an underrepresented population in the genetic counseling field, it is not known which aspect of diversity (e.g., ethnicity, LGBTQ, gender) they represented. Finally, over half of the respondents completed their undergraduate degree over five years ago. The amount of time that passed between completing the GCCP and participating in this survey may have impacted the ability of these respondents to accurately reflect on their experience with the program.

In summary, the Rutgers GCCP is a unique form of undergraduate genetic counseling exposure that is advantageous for several reasons, ranging from its ability to familiarize students with the profession to its ability to introduce underrepresented populations to the field. Moving forward, the GCCP can enhance exposure by educating its students about the non‐traditional roles available to genetic counselors. Implementation of programs like the GCCP may be an option for institutions looking to add to the amount of genetic counseling exposure offered to their undergraduate students. Creating more GCCP‐like programs could contribute to national professional efforts to increase diversity within the field. Future studies should investigate the challenges institutions may face when trying to implement additional exposures and explore ways these challenges can be overcome.

## AUTHOR CONTRIBUTIONS

E.M contributed to the design of the study, performed data collection, performed data analysis, and drafted the manuscript. J.R. contributed to study design, performed data collection, supervised data analysis, and contributed to the manuscript. M.H. contributed to the design of the study, supervised data analysis, and contributed to the manuscript. G.A.H. conceived and designed the study, supervised data analysis, and contributed to the manuscript. All authors have approved the final manuscript.

Erin McGraw, Jessica Rispoli, Michele B. Horner, and Gary A. Heiman confirm that they had full access to all the data in the study and take responsibility for the integrity of the data and the accuracy of the data analysis. All of the authors gave final approval of this version to be published and agree to be accountable for all aspects of the work in ensuring that questions related to the accuracy or integrity of any part of the work are appropriately investigated and resolved.

## COMPLIANCE WITH ETHICAL STANDARDS

### Conflict of interest

Erin McGraw, Jessica Rispoli, Michele B. Horner, and Gary A. Heiman declare that they have no conflict of interest.

### Human studies and informed consent

This study was reviewed and granted an exemption by the Rutgers University Institutional Review Board (Protocol: Pro2020002675). All procedures followed were in accordance with the ethical standards of the responsible committee on human experimentation (institutional and national) and with the Helsinki Declaration of 1975, as revised in 2000. Implied informed consent was obtained for individuals who voluntarily completed the online survey and submitted their responses.

### Animal studies

No non‐human animal studies were carried out by the authors for this article.

### Data sharing and data accessibility

The data that support the findings of this study are available from the corresponding author upon reasonable request.

## Supporting information

Supplementary Material
