# Supplementary material for: Genetic counseling certificate program: A program evaluation of undergraduate exposure to genetic counseling
Source: J Genet Couns. 2022 Feb 22;31(4):1003–7. doi: 10.1002/jgc4.1564 (PMC9541149; doi:10.1002/jgc4.1564)
Supplement: Supplementary file 1 — Supplementary Material [file JGC4-31-1003-s001.pdf]

Supplementary Material A: Student comments

Student 1. "The GCCP rotation gave me the closest possible experience to working as a genetic counselor. It helped me to feel familiar and comfortable with the scope of work that a genetic counselor does, to understand how genetic counselors interact with their colleagues, to see what goes on behind-the-scenes in between patient visits, and to get a sense of what a 'typical day' really looks like. I found tremendous value in being able to follow patients through multiple stages in their journey. In addition, I enjoyed seeing how each genetic counselor responded to patients and how their counseling style and strategy changed depending on the person and their needs. Lunchtime conversations with the genetic counselors were opportunities to ask questions about any and all aspects of the career and its ups and downs. I would never have had this insider's view of a genetic counseling career without the GCCP!"

Student 2. "I think the most beneficial aspect of the program is the opportunity for shadowing. In both my personal experience and discussions with classmates in my mater's [*sic*] program it was clear this was the hardest experience to find when learning about genetic counseling. In my shadowing I got to see several cases that gave me a stronger understanding of what it means to be a genetic counselor, including the prep and post visit work involved in the process."

Supplementary Material B: Survey questionnaire

1.) Do you consider yourself part of an underrepresented population in the genetic counseling field (ex. Hispanic, LGBTQ, male, etc.)?
    a. Yes
    b. No
2.) How long has it been since your undergraduate graduation?
    a. 0 – 5 years
    b. Greater than 5 years
3.) The following are the main objectives of the Genetic Counseling Certificate Program (GCCP). Please indicate how successful the program was at meeting these objectives by rating each on a scale of 1 - 5 (1 – strongly disagree, 2 – disagree, 3 – neutral, 4 – agree, 5 – strongly agree).
    a. The program enhanced my understanding of the genetic counseling profession.
    b. I gained experience talking to individuals who were in a crisis.
    c. After completing the program, I had a good understanding of the requirements that needed to be met to apply to genetic counseling graduate programs.
    d. I gained genetic counseling experience in a clinical setting.
4.) Add any comments that are relevant to the main objectives of the GCCP.
5.) Rate the following on a scale from 1 – 5 (1 – strongly disagree, 2 – disagree, 3 – neutral, 4 – agree, 5 – strongly agree).
    a. I would recommend this program to undergraduate students interested in pursuing a career in genetic counseling.
    b. The amount of time I was required to commit to this program was appropriate.
    c. My decision to apply/not apply to genetic counseling graduate programs was impacted by my participation in the certificate program.

d. Overall, I would consider my participation in the certificate program a positive experience.

6.) What was the most beneficial aspect of the program?

7.) What improvements would you suggest?

8.) Please chose one of the following:

a. I chose to apply and was accepted into a genetic counseling master's program.

b. I chose to apply and was not accepted into a genetic counseling master's program.

c. I did not apply to a genetic counseling master's program.

For those who chose to apply and were accepted to a genetic counseling master's program:

9.) Did participating in the certificate program effectively prepare you for the application process?

a. Yes

b. No

10.) For those who answered yes to #9, how did the certificate program prepare you for the application process? Please check all that apply.

a. The program ensured that I had completed the pre-requisite courses required by masters-level programs.

b. The clinical rotation I was required to complete gave me a better understanding of the genetic counseling profession, which I was able to discuss in my application/during my interview.

c. I was able to talk about counseling experience in my application/interview because the GCCP required me to volunteer at a crisis hotline or something similar.

d. I was given the opportunity to refine my interview skills in mock interviews.

e. Other

i. Please specify

11.) When did you first apply to graduate schools?

a. As a senior in college
b. After taking a gap year
c. Other
    i. Please specify

12.) Did your participation in this program impact when you decided to apply?

a. Yes
b. No

13.) For those who answered yes to #12, please explain how your participation in the program impacted when you decided to apply.

14.) How many times did you apply prior to your acceptance?

a. 1
b. 2
c. 3+

15.) Did you feel participating in the certificate program was advantageous during your time as a graduate student?

a. Yes
b. No

16.) For those who answered yes to #15, why was your participation in the GCCP advantageous during your time as a graduate student? Please check all that apply.

a. My participation in the GCCP provided me with a good baseline understanding of the genetic counseling profession.
b. Having the opportunity to rotate with genetic counselors for a semester familiarized me with some of the basic skills counselors regularly use (ex. taking a family history).

c. The required crisis volunteering experience taught me counseling techniques that could be applied to the genetic counseling profession.

d. Other

i. Please specify

17.) If given the opportunity, as a practicing genetic counselor, would you choose to work with students participating in the certificate program or something similar?

a. Yes

b. No

For those who chose to apply and were not accepted to a genetic counseling master's program:

9.) When did you first apply to graduate schools?

a. As a senior in college

b. After taking a gap year

c. Other

i. Please specify

10.) Did your participation in this program impact when you decided to apply?

a. Yes

b. No

11.) For those who answered yes to #10, please explain how your participation in the program impacted when you decided to apply.

12.) How many times did you apply?

a. 1

b. 2

c. 3+

13.) Do you plan on applying again?

a. Yes

b. No

14.) If you reapplied or are planning to reapply, did participating in the certificate program impact this decision?

a. Yes

b. No

c. Not applicable

<u>For those who chose not to apply to a genetic counseling master’s program</u>

9.) Do you think you would have applied had you not participated in the certificate program?

a. Yes

b. No

10.) Which career path did you decide to pursue?

11.) Did participating in the certificate program influence the career path you chose to pursue?

a. Yes

b. No

12.) For those who answered yes to #11, how did participating in the GCCP influence the career path you chose to pursue?
